# Peripheral Edema associated with Olanzapine: case report

**DOI:** 10.1192/j.eurpsy.2023.439

**Published:** 2023-07-19

**Authors:** H. Jemli, Y. Zgueb, R. Zaibi

**Affiliations:** ^1^Psychiatry deparment A, Razi Hospital, Manouba, Tunisia; ^2^Psychiatry deparment A, Razi Hospital, Manouba

## Abstract

**Introduction:**

Olanzapine is a second generation antipsychotic. Sedation and weight gain are common treatment side effects. However, other rare side effects such as peripheral edema are yet to be documented.

**Objectives:**

Our study aimed to describe the clinical presentation of edema in a patient taking Olanzapine.

**Methods:**

Case report

**Results:**

We present the case of a 42 male patient hospitalized for a manic episode. He was put on Olanzapine at 10 mg a day. During the hospitalization, the patient exhibited profuse pitting edema on his lower limbs and a rapid weight gain. He presented no other physical sign such as a fever, cutaneous lesions or trouble walking. Thrombophlebitis and erysipelas were eliminated after an extensive physical exam, complete blood work and doppler ultrasound exam of both legs.

Olanzapine was discontinued and the patient was prescribed a 4-day course of loop diuretics. Complete resolution of symptoms was noted 5 days later.

**Conclusions:**

Further research regarding the mechanism behind edema in patients taking second generation antipsychotics are needed. We recommend monitoring for edema with initiation and titration of Olanzapine treatment.

**Disclosure of Interest:**

None Declared

